# Long-Term Hb_A1c_, Physical Fitness, Nerve Conduction Velocities, and Quality of Life in Children with Type 1 Diabetes Mellitus—A Pilot Study

**DOI:** 10.3390/healthcare8040384

**Published:** 2020-10-03

**Authors:** Katharina Schiller, Markus Kofler, Martin Frühwirth, Michaela Fantur, Markus Rauchenzauner

**Affiliations:** 1 Department of Pediatrics and Neonatology, Kliniken Ostallgäu-Kaufbeuren, 87600 Kaufbeuren, Germany; katharina.schiller@kliniken-oal-kf.de; 2 Department of Neurology, Hochzirl Hospital, 6170 Zirl, Austria; markus.kofler@i-med.ac.at; 3 Department of Pediatrics, Hospital St. Vinzenz, 6511 Zams, Austria; martin.fruehwirth@krankenhaus-zams.at (M.F.); michaela.fantur@krankenhaus-zams.at (M.F.); 4 Department of Pediatrics, Innsbruck Medical University, 6020 Innsbruck, Austria

**Keywords:** type 1 diabetes mellitus, children, Hb_A1c_, quality of life, metabolic control

## Abstract

Objective: The aim of this study was to examine a possible association of Hb_A1c_, quality of life (QoL), fitness, and electrophysiological parameters in children with type 1 diabetes mellitus (T1DM). Methods: The study population (n = 34) consisted of patients with T1DM (n = 17) and an age-, sex-, and BMI-matched healthy control group (n = 17). Hb_A1c_ was obtained from patients with T1DM at time of diagnosis (T0), at 6 months (T6), at 12 months (T12), and at time of study inclusion (Tstudy). QoL was determined with a standardized questionnaire (KINDL-R). All children completed a 6-min walk test (6MWT) to evaluate their fitness level. Electrodiagnostic studies established upper and lower limb motor and sensory nerve conduction velocities (NCV). Results: Higher Hb_A1c_ (Tstudy) was associated with lower QoL showing in the subscales self-esteem, friends, and school. Higher Hb_A1c_ at (T6) and (T12) was associated with lower QoL in the subscale self-esteem. Based on various subscales, perceived problem areas differed significantly between children and their parents. No differences in fitness level and NCV were found between patients and controls except for a significantly slower median motor NCV in patients. Hb_A1c_ was not associated with NCVs at this early stage of disease. Conclusions: Good metabolic control reflected by adequate Hb_A1c_ values seems to be important for a good QoL in children with T1DM. Early Hb_A1c_ might be associated with QoL during follow-up.

## 1. Introduction

One important goal of diabetes management in children and adolescents is to achieve psychological well-being and a high level of quality of life (QoL) despite chronic disease burden [1]. The association of metabolic control and QoL in patients with type 1 diabetes mellitus (T1DM) has been already shown in several studies [2,3]. Hoey et al. found that good metabolic control—indicated by lower Hb_A1c_ values—is associated with a better QoL in adolescents with T1DM [3]. Additionally, girls showed a poorer overall QoL than boys [3]. In the assessment of QoL, it seems important to separate ratings of children and their parents [4]. Ratings of adolescent QoL and burden perceived by parents were different [3].

Findings are mixed concerning the association of metabolic to nerve conduction abnormalities [5]. Peripheral neuropathy is one possible complication of T1DM and occurs more often with increasing duration of disease [6]. Additionally, it is mainly found in adult patients associated with increased morbidity and mortality [6]. Symptomatic peripheral neuropathy is uncommon in children, but nerve conduction studies demonstrated subclinical neuropathy in 28–58% of children with T1DM [7,8,9,10]. The progression of subclinical peripheral nerve conduction abnormalities is predicted by poor metabolic control and is associated with body height and enduring hypoglycemia [5,11]. Despite modern multiple insulin injection therapy enabling good metabolic control, children and adolescents with insulin-dependent diabetes may still show subclinical nerve dysfunction [12]. There is evidence that early deficits in nerve conduction predict the progress of diabetic neuropathy [13] enforcing the focus both on motivating children for metabolic control and on the early detection of children with nervous system abnormalities [14].

The fitness of children with T1DM is controversially discussed in the literature. Some studies suggest the fitness of children with T1DM is reduced compared to healthy controls [15]. Chronic hypoglycemia in patients with T1DM might lead to alterations in aerobic and anaerobic muscle functions, as assessed by maximal isometric grip strength and an incremental cycling test until exhaustion, respectively. Impaired muscle function was found in children with poor glycemic control, whereas children with good metabolic control did not show reduced fitness [16].

Findings in QoL, subclinical neuropathy, and fitness of children with T1DM are still under debate. Data are sparse concerning the association of long-term Hb_A1c_ and QoL later on. Therefore, the goal of this pilot study was to evaluate a possible association of metabolic control from the onset of disease over time, QoL, nerve conduction, and fitness in patients with T1DM compared to a healthy age-, sex-, and BMI-matched control group.

## 2. Materials and Methods

This single-center study was conducted at the Department of Pediatrics, Saint Vinzenz Hospital, Zams, Austria.

Patients with T1DM were recruited during outpatient visits. Children with other chronic diseases, genetic syndromes, or neurological disorders were excluded from the study. All participants had no abnormalities in motor and cognitive development. The subjects were ambulatory, normally physically active, and on no additional medication. Healthy children matched for age, sex, and body mass index (BMI) seen as outpatients for routine were selected as the control group.

**Figure 1:**
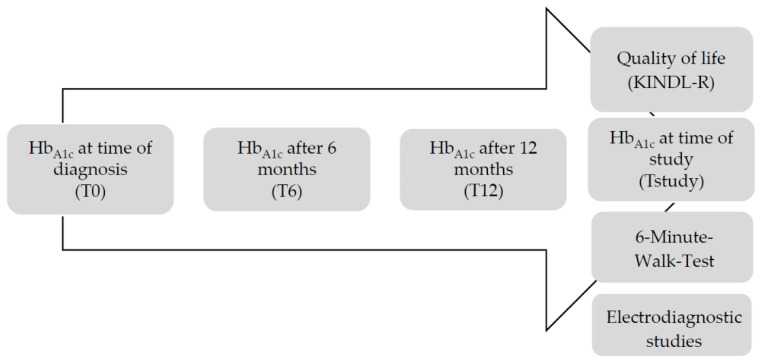
Standardized follow-up from time of diagnosis of T1DM until time of study inclusion.

In patients with T1DM, Hb_A1c_ values were obtained during outpatient visits. Both patients and healthy controls, and their parents, filled out separately a standardized QoL questionnaire. Each participant completed a 6-min walk test (6MWT) and underwent assessment of nerve conduction velocities (NCVs). In addition, weight and height of all participants were measured using a wall-mounted stadiometer and a calibrated weight scale. BMI was computed and converted to standardized BMI using the national BMI reference [17]. The standardized follow-up is presented in Figure 1.

The study protocol was approved by the local ethics committee (Medical University Innsbruck, AN5004 – session 323/4.11) and written informed consent was obtained from all patients, controls, and their parents.

### 2.1. Hb_A1c_ Measurements

Hb_A1c_ measurements of each patient were obtained retrospectively at time of diagnosis of T1DM (T0), at 6 months (T6), at 12 months (T12), and at time of study (Tstudy).

### 2.2. Quality of Life Questionnaire

The questionnaire “Kinder Lebensqualität Fragebogen” measuring QoL in children and adolescents (revised version KINDL-R) [18,19] was filled out by the children and one of their parents (proxy version). The KINDL is developed for children and adolescents between 3 and 17 years in age-specific versions, and there are disease-specific modules for chronic diseases (https://www.kindl.org). In this study, the German version for 7–13 years (children version), 14–17 years (children version), and 7–17 years (parent version) with the additional module for diabetes were used. The questionnaire consists of 24 items equally divided into six subscales: physical wellbeing, emotional wellbeing, self-esteem, family, friends, and school. The items measure the average feelings and experiences during the past week and are rated on a five-point scale (from 1 = never to 5 = always). Mean item scores of all subscales and the total QoL score were calculated and transformed to a scale ranging from 0 to 100 with 100 representing the highest QoL.

### 2.3. 6-Minute Walk Test (6MWT)

Each study participant completed a 6MWT to determine the personal level of fitness according to the guidelines of the American Thoracic Society as previously published and modified for children [20]. Before and after the walk, heart rate was measured with a finger pulse oximeter (Nonin Flight Stat, Aeromedix, Jackson, MI, USA).

### 2.4. Nerve Conduction Velocity (NCV)

Objective, sensitive, and validated measure of nerve function is the assessment of NCV [21]. Surface electrodes were used for assessing nerve conduction with standard technique. Motor conduction velocities were measured unilaterally in the median, ulnar, peroneal, and tibial nerves. Sensory conduction velocities were measured unilaterally in median, ulnar, and sural nerves.

The electrophysiological recordings were evaluated by two independent raters and discrepancies were resolved through discussion.

### 2.5. Statistical Analysis

Statistical Package for Social Sciences for Windows (SPSS Inc., Version 15.0) was used for the statistical analysis. 

Due to the small sample size nonparametric tests were chosen. Group differences were assessed using the Mann–Whitney U test and correlation of metric variables was analyzed with Spearman correlation. Data presented are the mean and standard deviation (SD). Statistical tests were performed two-tailed with an alpha level of < 0.05 indicating statistical significance.

## 3. Results

Thirty-four participants were eligible, and all agreed to participate in the study. All patients were included in statistical analyses. Subjects were grouped in 17 patients with T1DM (6 girls, 11 boys) and 17 controls (6 girls, 11 boys).

Demographic data and clinical characteristics are presented in Table 1.

### 3.1. Quality of Life (QoL)

There were no group differences between patient and control groups for the total QoL score or with any of the child-rated and parent-rated subscales. No sex differences were found. Total QoL and subscale values are presented in Table 2.

Hb_A1c_ (T0) was neither correlated with the total score of QoL nor with any subscales child-rated or parent-rated.

Both, Hb_A1c_ (T6) and Hb_A1c_ (T12) were inversely associated to QoL subscale “self-esteem” child-rated (r = −0.73, *p* = 0.005; r = −0.56, *p* = 0.037, respectively).

Hb_A1c_ (Tstudy) was inversely associated to the total score of QoL parent-rated (r = −0.53, *p* = 0.018) and in particular to the following subscales: “self-esteem” child-rated (r = −0.69, *p* = 0.003), “friends” parent-rated (r = −0.56, *p* = 0.012) and “school” parent-rated (r = −0.78, *p* = 0.001).

### 3.2. Anthropometric Parameters and 6-Minute Walk Test (6MWT)

Patients and controls did not differ significantly in anthropometric parameters, walking distance (6MWD), and heart rate (pre/post walking) of 6MWT as presented in Table 3.

The 6-min walk distance (6MWD) was not associated with QoL and any subscales; 6MWD and heart rate were not correlated with Hb_A1c_ (Tstudy) in the patient group.

### 3.3. Nerve Conduction Velocity (NCV)

NCVs are presented in Table 4. Patients and controls did not differ significantly except for a significantly slower median motor NCV in patients. Correlation between Hb_A1c_ (Tstudy) and NCVs did not reach statistical significance.

Over all analyses, there were no significant differences in patients with T1DM using an insulin pump versus patients using no insulin pump. Additionally, there were no sex differences.

## 4. Discussion

The most important finding of the present study is that Hb_A1c_ obtained during the first year after diagnosis of disease is inversely correlated to certain subscales of QoL of patients with T1DM at Tstudy, i.e., some 5 years after disease onset. Our results are therefore in line with previous studies in which good metabolic control was shown to be associated with better QoL [3]. The ratings of parents and children differed from each other as already found by Hoey et al. [3,4] enforcing the importance of separate ratings.

In previous studies, Hb_A1c_ was measured at time of study inclusion, e.g., more than 5 years after diagnosis (3), whereas in the present study, Hb_A1c_ was obtained during the first year after diagnosis of disease. The development of Hb_A1c_ from onset of T1DM over a year was found to be correlated with QoL at Tstudy, i.e., 4.9 (3.6) years after onset. When Hb_A1c_ was higher in the first year, patients reported significantly lower QoL on average 4 years later. These results concur with Hb_A1c_ being associated with QoL during follow-up. Thus, the adjustment of metabolic control reflected by Hb_A1c_ from onset of T1DM might have an impact on the well-being of the children later on. Importantly, patients indicated lower self-esteem. As low self-esteem is associated with psychiatric disorders such as depression or substance use [22,23], children with T1DM might need close follow-up. Alternatively, Hb_A1c_ could as well be the reflection of QoL, or at least could be the result of lifestyle, that in turn also can correlate with the QoL. Therefore, the association of QoL and Hb_A1c_ found in this pilot study does not allow us to draw assumptions regarding causality, as either one might be a confounder for the other one.

The development of Hb_A1c_ after disease onset varied during the observational period. Hb_A1c_ was highest at T0. At T6 the decline in Hb_A1c_ is probably due to a more rigorous adjustment of metabolic control. Later on, the motivation of children is likely to be reduced as indicated by an increase in Hb_A1c_ at T12. This underlines the importance to keep the focus on good metabolic control and on the acceptance of the disease in order to enhance QoL also later on.

With respect to Hb_A1c_ (Tstudy), the QoL ratings of parents and patients differed. Children with higher Hb_A1c_ values rated themselves lower on overall QoL especially on the subscale of self-esteem, whereas parents perceived lower QoL of their child on the subscales of friends and school. This is an important issue for diabetes management because the different perceptions of patients and parents may call for the need of tailored support in order to discover problem fields and to maximize QoL. Furthermore, QoL might be influenced significantly by sociodemographic variables such as economic class, as shown by de Souza et al. [24]. Since sociodemographic information was not evaluated in this study, a confounding effect might be possible.

The final part of the study was to measure fitness level and electrophysiological abnormalities in children with T1DM. As patients and controls did not differ significantly in the results of the 6MWT the subjects were presumably on average at the same fitness level. This is in contrast to previously published findings of reduced fitness in children with T1DM [15]. Notably, only children with poor metabolic control showed alterations in aerobic and anaerobic muscle functions [16].

For electrodiagnostic parameters, a detailed neurophysiological examination of children with T1DM compared to healthy children was performed. There were no electrophysiological abnormalities in patients with T1DM compared to control group, except for a significantly slowed median motor NCV. Additionally, no correlation between NCVs and Hb_A1c_ was found at any point in time. This is in contrast to other studies reporting frequent subclinical neuropathy in diabetic children [13,14]. To us, due to the small sample size, effects might have not reached statistical significance except for the slower median motor NCV. Additionally, small discrepancies between the two raters that were resolved by discussion might have influenced the effect. Furthermore, no correlation between NCVs and Hb_A1c_ was found at any point in time. Notably, mean disease duration was considerably longer in previous studies, exceeding seven years [14]. Diabetic polyneuropathy did therefore likely not occur at this early stage of disease in the present study. Nerve conduction studies are the gold standard for the detection of subclinical neuropathy and determining neurophysiological measurements [15]. Measuring NCVs in children is a big challenge, which renders it often difficult to find differences in T1DM patients. Due to artefacts and limited compliance of the children, a supramaximal stimulation was not always ensured in our study. Potentially, more suitable screening tools such as vibration sensation thresholds and thermal discrimination thresholds that are quicker and easier in the implementation might be more appropriate for use in studies of children [14].

The main limitation of the study is the small sample size. Therefore, the findings might not represent the larger population. Further research with a larger sample size with equal gender sizes and a second experimental group with children showing a well-controlled T1DM is needed. Additionally, the influence of sociodemographic data on QoL seems to be an important factor to be addressed in the future. Another limitation of this pilot study is that despite an association of QoL and Hb_A1c_ was found, no assumptions regarding causality can be drawn. As either Hb_A1c_ could be a confounder of QoL, or vice versa, that puzzle is difficult to solve, particularly with the small sample size.

## 5. Conclusions

In this comprehensive pilot study, children with T1DM showed no significant clinical or subclinical differences to healthy controls with regard to fitness level and neurophysiological abnormalities. However, there was an association of Hb_A1c_ at T(6), T(12), and T(study) with subscales of the “Kinder Lebensqualität Fragebogen” measuring QoL. It seems inevitable—from the onset of disease—to teach patients with T1DM about the importance of good metabolic control, which might be connected to a better QoL during follow-up. Regarding the disease management and according to the different perceptions of parents and their children, the focus needs to be more on the children and teenager to ensure parent perspectives do not miss important clinical and psychological cues from the child.

## Figures and Tables

**Table 1:** Baseline characteristics of the participants.

		Patients (n = 17)	Controls (n = 17)
**Age in years**	mean (SD)	13.3 (3.8)	13.5 (3.8)
**Sex, female/male**		6/11	6/11
**Disease duration in years**	mean (SD)	4.9 (3.6)	
**Hb_A1c_% (T0)**	mean (SD)	8.6 (0.9)	
**Hb_A1c_% (T6)**	mean (SD)	7.0 (0.9)	
**Hb_A1c_% (T12**	mean (SD)	7.6 (0.9)	
**Hb_A1c_% (Tstudy)**	mean (SD)	7.9 (1.4)	
**Total daily dosage insulin/kg**	mean (SD)	0.8 (0.2)	
**Insulin pump (yes/no)**		10/7	

**Table 2:** Total quality of life (QoL) and subscales measured with KINDL-R child-rated and parent-rated in the two study groups. Values are means (standard deviation).

	Patients (n = 17)		Controls (n = 17)	
	Child-Rated	Parent-Rated	Child-Rated	Parent-Rated
**Total QoL**	80.5 (8.6)	80.1 (9.5)	79.9 (11.8)	75.7 (12.0)
**Subscale physical wellbeing**	78.6 (11.9)	81.3 (14.1)	67.9 (22.6)	70.7 (25.5)
**Subscale emotional wellbeing**	86.1 (12.4)	82.0 (13.9)	84.6 (15.5)	78.5 (17.9)
**Subscale self-esteem**	71.4 (13.6)	75.8 (15.1)	79.3 (14.8)	70.3 (15.2)
**Subscale family**	85.7 (9.6)	81.3 (12.1)	87.9 (16.1)	79.7 (17.9)
**Subscale friends**	85.2 (11.4)	81.1 (9.9)	77.5 (17.9)	78.9 (14.8)
**Subscale school**	68.3 (18.7)	79.0 (19.4)	79.0 (20.7)	75.8 (19.7)

**Table 3:** Anthropometric parameters and 6MWT showing standardized (SDS) height, weight, BMI, 6MWD (meters), and heart rate (beats per min). Values are means (standard deviation).

		Patients (n = 17)	Controls (n = 17)	*p*-Value
**Height SDS**		0.1 (0.8)	0.7 (1.2)	*p* = 0.231
**Weight SDS**		0.1 (0.7)	0.7 (1.1)	*p* = 0.339
**BMI SDS**		0.1 (0.8)	0.5 (1.2)	*p* = 0.245
**6MWD**		639.4 (110.5)	649.4 (60.0)	*p* = 0.929
**Heart rate**	pre-walk	90.5 (20.2)	85.8 (17.2)	*p* = 0.423
	post-walk	144.1 (37.5)	148.5 (23.5)	*p* = 0.323

**Table 4:** Nerve conduction velocities (m/s) in the two study groups. Values are means (standard deviation).

	Patients (n = 17)	Controls (n = 17)	*p*-Value
**Motor nerves**			
Median	53.1 (3.2)	58.5 (5.1)	*p* = 0.006
Ulnar	52.7 (3.8)	55.0 (6.3)	*p* = 0.114
Peroneal	47.3 (4.6)	48.2 (5.1)	*p* = 0.316
Tibial	44.3 (7.9)	46.5 (6.0)	*p* = 0.186
**Sensory nerves**			
Median	54.6 (8.8)	57.8 (6.0)	*p* = 0.178
Ulnar	55.1 (9.9)	56.6 (8.6)	*p* = 0.245
Sural	48.3 (5.3)	47.5 (3.8)	*p* = 0.608
